# Adding Meaning and Context to Nodes and Edges: A Mixed Methods Approach to Enhance the Validity of Idiographic Networks

**DOI:** 10.1007/s10608-025-10670-6

**Published:** 2025-11-10

**Authors:** Melissa Miléna De Smet, Lune Imaani Quintens, Dries Debeer, Joran Jongerling

**Affiliations:** 1 Tilburg University, Tilburg, Netherlands; 2 Ghent University, Ghent, Belgium

**Keywords:** Experience sampling methods (ESM), Idiographic networks, Personalized care, Mixed methods, Reflexive thematic analysis, Validity

## Abstract

**Background:**

Experience Sampling Methods (ESM) are increasingly used to derive idiographic networks, which promise more personalized interventions. Using the most frequently used network approach (VAR-model), we demonstrate the value of qualitative methods to enhance their validity.

**Methods:**

A multiple case study with four adolescents examined the relationship between identity/self-esteem and depressive complaints. Quantitative ESM data was enriched with open-ended ESM data and semi-structured interviews. In a mixed methods parallel design, VAR-based networks and reflexive thematic analyses were combined.

**Results:**

The qualitative data gave meaning and context to adolescents’ ESM data and networks, revealed uncaptured experiences and challenged associations in the network.

**Conclusion:**

Mixed methods approaches can enhance the validity of idiographic networks by personalizing ESM designs prior to assessment, adding meaning and context to nodes and edges, and providing alternative explanations for observed dynamics. We discuss several reasons, strategies and ways forward for mixed methods idiographic networks.

**Supplementary Information:**

The online version contains supplementary material available at 10.1007/s10608-025-10670-6.

## Introduction

Experience Sampling Methods (ESM), in which individuals are monitored several times a day for several days (e.g., via a smartphone app), have become the gold standard to assess person-specific dynamics (e.g., related fluctuations in mood and affect). These dynamics can be represented in an idiographic network, consisting of nodes (that represent constructs of interest, e.g., emotions) and edges (that represent relationships between constructs). Such ESM-based idiographic networks promise more than mere descriptive feedback on how a patient is progressing. Because they present associations among variables (e.g., symptoms, emotions, cognition, and behavior) over time (Bringmann et al., 2022a), the expectation is that potential interactions can be identified that can guide the intervention. For instance, if a temporal relationship is found between self-image and sadness within an adolescent, the intervention could aim at improving the adolescents’ self-image to resonate changes in their mood (Borsboom & Cramer, 2013). ESM-based idiographic networks have become today’s hope for personalized interventions (e.g., Roefs et al., 2022).

However, the validity and clinical utility of ESM and the resulting idiographic networks present ongoing challenges (see Bringmann et al., 2022b, 2024; De Smet et al., 2024; Stone et al., 2023). No guidelines exist to ensure that idiographic networks indeed offer a useful guide for personalized care. In this paper, we take patients’ perspectives as our reference point. Using a ‘validation through triangulation’ approach, qualitative research methods are employed to enhance the validity of ESM-based idiographic networks (as suggested by De Smet, 2024; De Smet et al. (2024).). The value of qualitative methods for clinical applications has been demonstrated for idiographic symptom networks (Van den Bergh et al., 2024). Some other pioneering developments and examples can be found in the work by Scholten et al., (2022) on integrating theory-based and data-driven methods to case conceptualization, Klintwall et al. (2023) on perceived causal networks, and the mixed methods case study by Hulsmans et al., (2024a, 2024b).

The currently most widely used method to infer idiographic networks from intensive longitudinal data is the vector autoregressive (VAR) model. In contrast to cross-sectional networks, VAR-based networks explicitly represent relationships between constructs over time and are person-specific (for a detailed explanation see Bringmann et al., 2024). The statistical and theoretical limitations of VAR-based models (and networks in general) are subject of an ongoing discussion and beyond the scope of the present paper (see Bringmann et al., 2024; Oude Maatman & Eronen, 2025). Here, we use them as an illustration of common practices. Departing from a critical clinical-methodological position, we consider statistical networks as constructed rather than real representations of patients’ psychopathology, which *could* stimulate clinically conversations, *if* they manage to capture meaningful things.

### A Mixed Methods Approach to Enhancing Valdity: Adding Meaning and Context

Thus, to realize personalized care, further clinical validation of both idiographic measurement (i.e., ESM) and the resulting dynamics (i.e., VAR-based networks) are needed. To this end, we propose to enrich quantitative (e.g., ESM; questionnaires) with qualitative methods (e.g., open answer options; interviews). By inquiring patients’ subjective experiences, the personal meaning of nodes (e.g., emotions) can be clarified (e.g., crying is relieving). By gaining rich descriptions on patients’ daily lives, potential explanations for why certain interactions are observed can be generated (e.g., with whom, when and where do emotions occur), adding clinically relevant insights. By triangulating idiographic assessment (currently predominantly quantitative) with idiographic experiences (qualitative), results can be cross-validated and in-depth understanding is added to abstract statistical results.

Triangulating idiographic networks and qualitative analyses, three questions can be asked: how do the quantitative and qualitative strands 1) converge, 2) diverge and 3) complement each other? The first question examines how one strand corresponds to the observations of the other. The second question addresses the opposite: where does one method challenge or contrast the conclusions drawn by the other? The third question asks if the strands add knowledge which would not have been picked up if we would have relied on only one of them. Although related, distinguishing these questions is important. While the first and second question depart from the assumption that the same phenomenon is being observed, yet with different measures, the third question assumes that distinct yet valid knowledge can be obtained by using different methods. As such, methodological triangulation is a powerful way to enhance the validity of observations and analyses, enriching observations and substantiating conclusions (Hesse-Biber, 2010).

In this paper we take adolescent depression as a critical example. We use a mixed methods parallel design to study person-specific relations between adolescent depression and identity. We 1) estimate idiographic VAR models on the (associations between) daily fluctuations in identity/self-esteem experiences and depressive symptoms, 2) inquire adolescents’ own identity experiences and what they find important in their daily lives, and 3) study how adolescents’ idiographic networks relate to their subjective experiences.

## Method

**Fig. 1:**
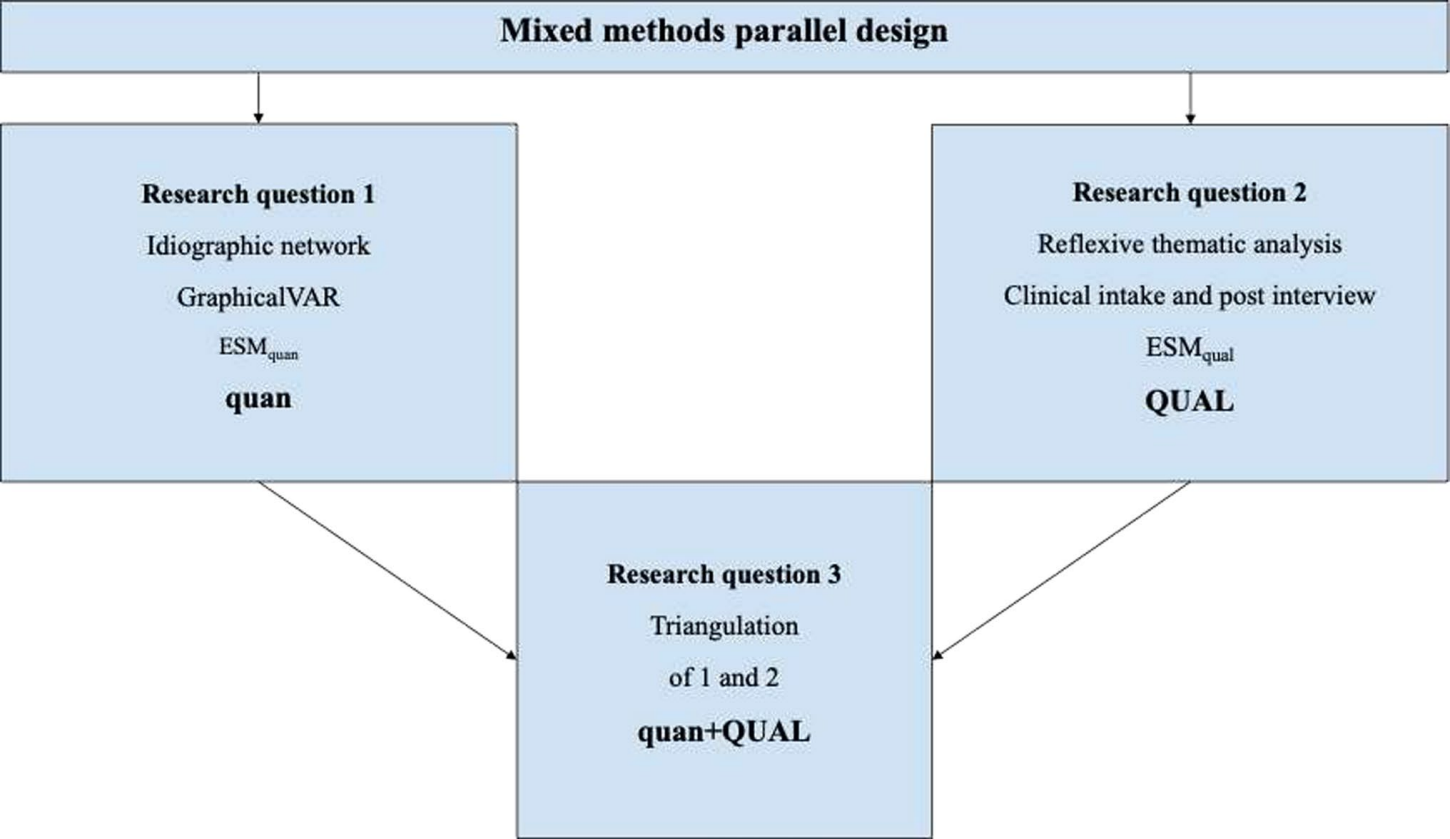
Mixed methods parallel design

A mixed methods multiple case study was conducted with four adolescents in a clinical setting. Using a parallel design (Giddings & Grant, 2006), the quantitative and qualitative strand ran simultaneously. The main emphasis of this study was on the qualitative strand (quan + QUAL). The integration of both strands occurred during the interpretation phase in which results from both strands were triangulated. This allowed for a cross-validation of the idiographic networks using the qualitative results and provided a more comprehensive perspective using complementary methods. Due to the parallel structure of design, the extent to which results from one strand could inform the other (e.g., for further data-gathering and analyses) was limited (in contrast to a sequential design; see Hesse-Biber, 2010). For each of the four adolescents in this study, a VAR-based idiographic network was combined with an inductive thematic analysis of their qualitative data. Given the broad and exploratory nature of this study, all available qualitative data was included to provide sufficient contextualization for each case. The design is depicted in Fig. 1.

### Ethics

This study was approved by the Ethics Committee of Ghent University Hospital (Belgium: BC08405). All participants signed informed consent prior to participation. Data-processing was performed under the European General Data Protection Regulation (GDPR, 2016/679).

### Setting

The study took place in a naturalistic community-based ambulant therapy setting in Flanders (Belgium; www.tejo.be) and is part of a larger mixed methods idiographic study on adolescent mental health and psychotherapy (De Smet, 2024). The therapeutic institution provides short-term solution focused therapy of ten weekly sessions to adolescents (from the age of ten). The therapy setting aims at low threshold care by providing immediate therapy (i.e., walk-ins, no or short waiting list), free of charge, and with limited administration (i.e., first name and phone number). Adolescents also have the choice whether or not to inform their caretakers about their therapy (i.e., discretion). As such, they serve as a community-based institution in the mental health care field.

### Participants

Participants were selected from a larger study conducted in a naturalistic community-based ambulant therapy setting (see De Smet et al., 2024). Four cases were purposefully selected based on the fluctuations in their ESM data and richness of their qualitative data. Cases with no more than 50% missingness (out of 120 momentary assessments; 20 days, 6/day), were considered. One exception was made (Kobe) because of the richness of the qualitative data (see Table 1). All four participants were given a pseudonym. All had a Belgian nationality; Table 1 provides further characteristics.

**Table 1:** Overview of characteristics per participant

	Jan	Ella	Sam	Kobe
Gender	Male	Female	Non-binary	Male
Age range	15–20	15–20	20–25	15–20
DSM diagnoses (SCID-5)	Depression, Bulimia	Depression in remission	Depression, ADHD*, ASD*, OCD, Separation disorder, Panic disorder, Social anxiety disorder, Agoraphobia	Depression in remission, Generalized anxiety disorder
Relationship status	In a relationship	Single	In a relationship	In a relationship
Number of siblings	2	2	1	2
Enrolled in higher education	Yes	Yes	Temporarily not	Yes
Highest education level mother	Unspecified	University	High school	University
Highest education level father	High school	University	High school	University
Previous mental health care	No	No	Yes	No
Medication	No	No	Yes	No
# Datapoints ESM (% completed) Momentary Morning Evening	83 (69,17%) 9 (45%) 16 (80%)	90 (75%) 7 (35%) 20 (100%)	74 (61,67%) 16 (80%) 20 (100%)	41 (34,17%) 7 (35%) 12 (60%)

*The diagnoses of ADHD and Autism Spectrum Disorder (ASD) for Sam were not assessed by the researchers, but adopted from the diagnostic assessment the participant undertook in previous clinical care

### Procedure and Instruments

**Fig. 2:**
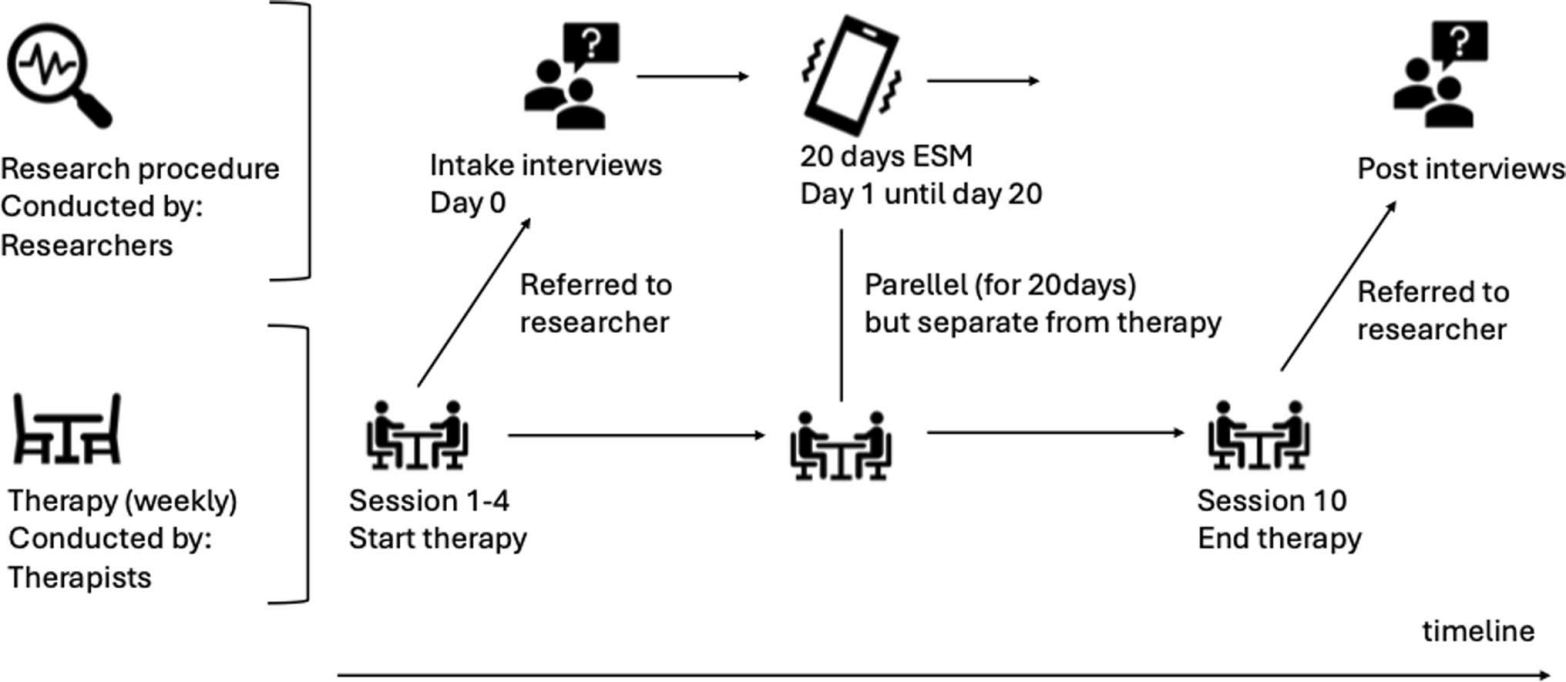
Study procedure

Figure 2 visualizes the study procedure. The research ran in parallel with therapy and was designed to interfere as little as possible with the treatment. The intake was therefore held at the start of participants’ therapy (i.e., < 5 therapy sessions) but never before a first session. Data were not shared or discussed with the therapist and the youth and were only collected for research purposes. Audio recordings of the interviews were transcribed and pseudonymized for the analyses. For the ESM part of the study, participants completed the ESM diary for 20 consecutive days, starting the day after the intake interviews. The EthicaData (Avicenna) app was used for data collection (https://avicennaresearch.com/).

#### Interviews

A *semi-structured interview* was conducted to understand how adolescents view themselves (self-evaluation of identity). The *post-interview* inquired about participants’ experienced changes since the start of therapy. The interview guides can be found on OSF (https://osf.io/nvz5r).

#### ESM diary

The conceptualization of the adolescent depression items was based on a literature review and commonly used DSM definition (e.g., Mullarky et al., 2019; Thapar et al., 2022). Based on this, relevant depression items were selected from the ESM Item Repository (https://www.esmitemrepository.com/), which were then reviewed by four student assistants and adjusted based on their feedback. The final set comprised 34 items. Response time was around two minutes. The momentary questionnaire remained available for 60 min and did not overlap with the next measurement. Morning and evening questionnaires remained available for 300 and 240 min respectively. A one-week pilot was conducted with ten age-relevant undergraduates, showing > 80% compliance.

### Data Analysis

#### Descriptive and Statistical Analysis

First, rows containing missing data were removed from the ESM dataset using listwise deletion, which is a common approach to handling missing data for VAR-based analyses using *GraphicalVAR* (Epskamp et al., 2018a; Epskamp, 2024). Second, descriptive statistics, including the mean, standard deviation and individual plots for each ESM item were computed in R (Version 4.2.2) using the *Lattice* package (Version 0.21; Sarkar, 2023). To allow observation of possible individual differences in depressive experiences (as expected given the heterogeneity in symptoms, see Thapar et al., 2022), we chose to work with the individual depression and identity/self-esteem items rather than their compounds.

The R package *graphicalVAR* (Version 0.3) was used for modelling the ESM data. In line with the procedure of graphicalVAR, multilevel vector autoregressive (VAR) models were used, and unequal distances were not accounted for between beeps. In addition, due to the small sample size, the LASSO estimator (least absolute shrinkage and selection operator; Epskamp et al., 2018a) was used in conjunction with extended Bayesian information criterion (EBIC). For each of the four participants, a subject-specific graphical VAR (GVAR) model was estimated to derive temporal and contemporaneous networks (Epskamp et al., 2018b). More details on the analysis can be found in the Appendix of this paper; interested readers are referred to the OSF page for the materials and R scripts (https://osf.io/jgz7t/).

#### Thematic Analysis

Reflexive thematic analysis (Braun & Clarke, 2019) was conducted inductively on the clinical intake and post-interviews, and the open-ended ESM responses. Given the descriptive aim of this study and nature of the qualitative data gathered, thematic analysis was deemed most suited. The choice for a reflexive type of thematic analysis corresponds with the researchers’ aim of arriving at themes based on inductive qualitative analysis and the acknowledged role of the researchers’ subjectivity in this process (Braun & Clarke, 2019).

The analyses were carried out by the second author using Nvivo-14 software (Lumivero, 2023); the first author served as an auditor throughout the process. During the initial data familiarization phase, participants’ responses were repeatedly read. Meaningful sections were identified, and initial notes were made on recurring themes within each case. During a first open coding phase, we stayed close to the own words of the adolescents using in vivo codes. Next, initial codes were organized in clusters and further condensed into themes based on common meanings, following an iterative process. For internal coherence and external distinction, clear definitions were phrased for each theme. A preliminary version of the themes was discussed by the first and second author and subsequently revised. Themes were reviewed on their meaningfulness to capture each case’s experience. Themes were initially generated for the ESM and interviews separately (these can be found in the Appendix) and later integrated to form overarching themes per case. In a final step, central themes across the four cases were constructed.

#### Quality Control

We acknowledge the role of the researchers’ assumptions in the construction of the findings. With a background in clinical psychology and a main affinity with qualitative research, the first and second author (the coders) operated within a constructivist framework, however, influenced by post-positivist ideals. To prioritize subjective experiences over general diagnostic criteria, we stayed close to patients’ own words to construct themes. To minimize the impact of our own view and theoretical assumptions as much as possible, we took several measures. To ensure the credibility and trustworthiness of the analyses, transparency was prioritized throughout the research process. Investigator triangulation was employed to mitigate potential influences stemming from the researchers’ theoretical and clinical backgrounds. The overall team was balanced in qualitative and quantitative methodological expertise. A shared critical view on the possibilities of VAR-based networks for clinical applications motivated the execution of this study. Methodological triangulation (quantitative and qualitative methods) allowed for a critical comparison and a more comprehensive understanding. Data-triangulation (interviews and open-ended ESM) bolstered the credibility of the qualitative findings. The second author kept a reflective diary and used memos to document emerging ideas; the first author served as an auditor throughout. Regular meetings between the first and second author were held to review codes and themes, facilitating consensus-building and providing alternative perspectives. Likewise, for the analyses and interpretation of the idiographic networks, regular meetings were held between the first, second and third or fourth author. The triangulation of both strands was performed by the first and second author (Table 2).

**Table 2:** ESM items used in this study

Construct	ESM items	
	Momentary (6/day)	Answer scale
Depressive complaints	I am satisfied with how I look Just before the notification, I was worrying I am tired I feel sad	7-point Likert scale, ‘not at all’ (1) to ‘very much’ (7)
Identity (certainty)	I can be myself	
Self-esteem	I like myself I am ashamed of myself	
Activity	What were you doing before this notification?	Open-ended (text or voice recording*)
Important daily events and experiences	Morning (1/day)	
	What is the most important event that will happen for you today?	
	Evening (1/day)	
	What was the most positive event for you today? What was the most negative event for you today? Would you like to share more about your day and how you felt?*	

*Voice recordings were transcribed and pseudonymized prior to analysis

## Results

**Fig. 3:**
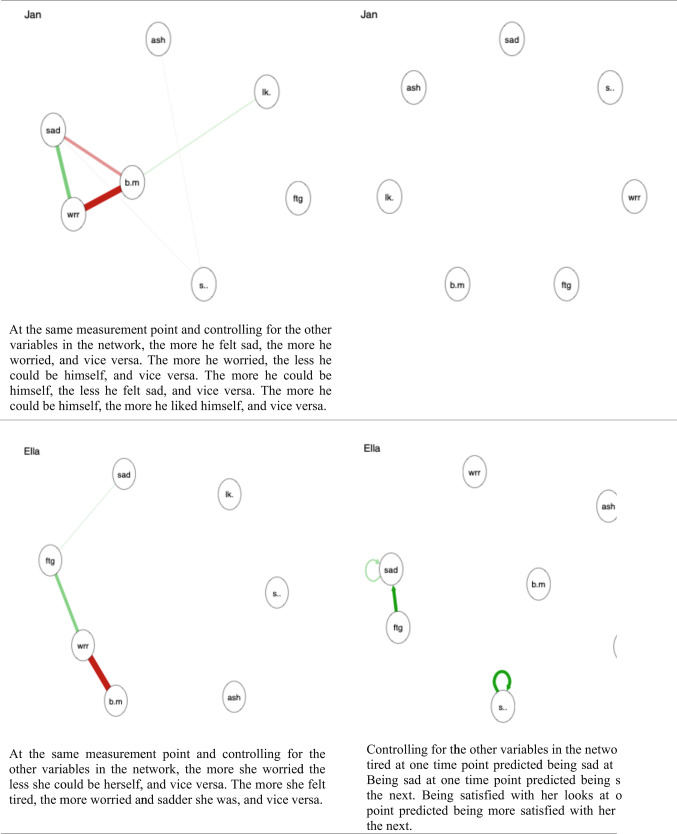

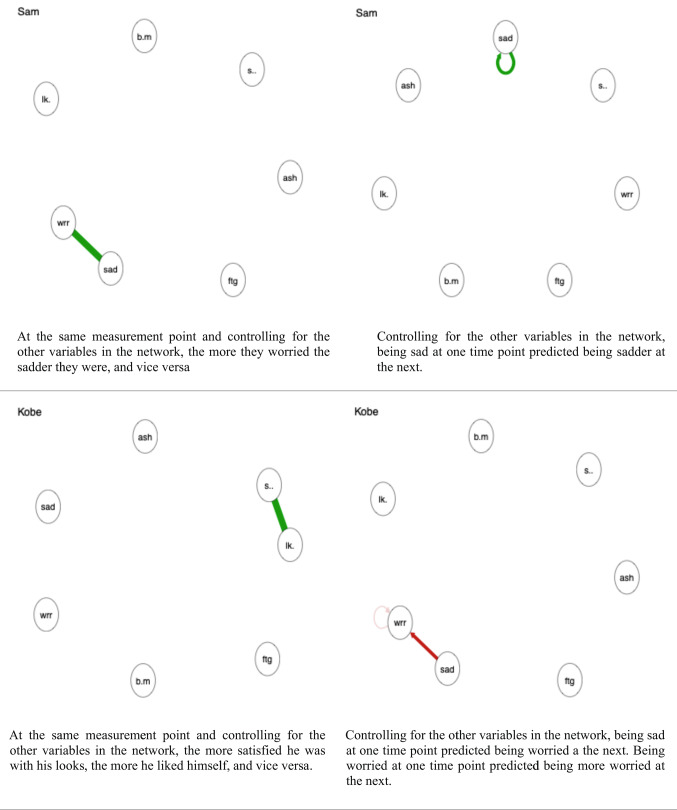
Partial Contemporaneous and Directed Correlation Networks per case. Note. sad: sadness; wrr: worrying; b.m.: be myself; ftg: fatigue; lk. like myself; s..: satisfied with looks; ash: ashamed. Partial contemporaneous correlation networks (left); partial directed correlation networks (right). Circles (nodes) represent variables of identity, self-esteem and symptoms of depression. Green edges indicate positive relationships; red edges indicate negative relationships. The thickness and saturation of the connections indicates the strength (absolute value) of the relationship. Only Sam’s networks proved reliable; interpreting the others’ networks must be done with caution

The parallel mixed methods study enriched idiographic VAR-based networks of four cases with their qualitative analyses of interviews and open-ended ESM data. Here, we present the integrated findings across all cases to answer the research questions: 1) which associations between daily fluctuations in identity experiences and depressive symptoms are identified within an adolescent’s network, 2) how do adolescents experience their identity and what they find important in their daily lives, and 3) how do the idiographic networks relate to the subjective experiences of the adolescent. Ways in which the quantitative and qualitative data converged, diverged and/or complemented each other are described. Figure 3 presents a visual summary of each participants’ networks and their interpretation. Note that only for Sam the networks could be interpreted reliably, the others are presented by means of illustration and must be regarded with caution. Table 3 provides on overview of the main themes across cases. More detailed results for each separate case can be found in the Appendix.

**Table 3:** Overview of main themes of identity experiences and what is important across cases

*Difficulty to be myself—with and due to others*	
Jan	Strong norms and morals, self-esteem depending on school and weight
Ella	A process of finding and expressing myself as being bisexual and non-religious, except with her mother
Sam	Recurring feelings of anxiousness, stress, depression and having problems with my weight Longing recognition for my Neurodivergence, Non-Binary Identity and Bisexuality A fear of being annoying and getting abandoned due to past experiences of bullying
Kobe	Feeling good when I’m productive, active and in control of my days but receiving little autonomy at home from parents Recurring arguments with significant others (girlfriend and parents)
* A need to share my emotions and be with the “right” people*	
Jan	The importance of being among people, away from conflicts at home The importance of being able to let go of emotions and share them with others
Ella	The importance of being in good company, like friends A transition from handling “mental fatigue” alone, while parents do not understand, to sharing my emotional side
Sam	The importance of having significant others close (mother, partner, cat)
Kobe	The importance of being able to share my problems with others
* Learning to prioritize myself through therapy*	
Jan	A process of finding mental peace, chosing for myself and my joy versus doubting my own choices
Ella	Learning to balance to do something for others versus self, with music being most important to me
Kobe	Slowly breaking out of the vicious cycle of self-doubt (e.g., looking fit remains important)

1. Adolescent-specific networks: associations between daily fluctuations in identity and depressive symptoms
  In the 20-day ESM data, fluctuations in identity and depressive symptoms were observed for each participant (see Table 1 in the Appendix). This resulted in case-specific dynamic networks for each case (see Fig. 3). For all but Sam, contemporaneous associations between depression and identity/self-esteem were observed; for none, directed relationships between identity/self-esteem and depression were found. However, only for Sam, these networks could be reliably interpreted, hence we limit the description to their results here (full details on all cases can be found in the Appendix). Interpreting the others’ networks must be done with caution (interpretations are added in Fig. 3 for illustration).
  Sam’s contemporaneous network (Fig. 3, left) visualizes a thick green edge between ‘I feel sad’ (M = 2.4; SD = 0.9) and ‘Just before the notification, I was worrying’ (M = 3.4; SD = 1.3), indicating a positive association (PCC = .08) controlling for other variables in their network. This indicates that whenever Sam reported to feel sad, they concurrently reported to worry. Sam’s temporal network (Fig. 2, right) indicates a thick green arrow for ‘I feel sad’, indicating a positive autoregression (PDC = .20). This suggests that when Sam reported feeling sad, they felt sadder at the next measurement point.
2. Adolescents’ experiences of identity and what they find important in their lives
  Across the four cases, highly unique themes were identified (see Table 3). Moreover, the ESM and interview data provided complementary insights into more micro and macro level processes respectively (see Table 2 in the Appendix). Across the four cases, three overarching themes were derived: 1) *Difficult to be myself—with and due to others,* 2) *A need to share my emotions and be with the “right” people,* and 3) *Learning to prioritize myself through therapy.* These themes are not mutually exclusive and must be understood as interrelated experiences.
  *Difficult to be myself—with and due to others*. First, all four participants struggled in different ways with their self-image and how to be themselves. This was expressed by a focus on wanting the ideal weight, good looks, hiding emotionality and having doubts about their gender identity, sexual orientation, religion, etc. For all four, these identity struggles came with feelings of insecurity, which they attributed to present (e.g., conflicts with parents) and past interpersonal experiences (e.g., being bullied).
  For instance, Jan’s difficulty to be or accept himself, was expressed in high moral standards and norms to pursue. For instance, school achievements gave him more energy, perseverance, and motivation, but he quit university during the exam period as his stress levels had become too high (post-interview). He noticed how school strongly impacted his self-esteem.
*“My self-esteem was better again, and motivation to do fun things again, and to get myself through it, and now with more stress I noticed that it has been pulled down again, that I needed to come here [therapy] again and tell my story again” (Jan, intake).*
  Similarly, a strong focus on his weight and physical appearance marked his self-esteem yet came at a high personal cost.
  *“My brother crashed… Without me realizing it, he had started a sort of crash diet, and I*
  *thought, if he can do it, so can I. I lost a lot of weight, which made me feel better at the time, but mentally worse and worse.” (Jan, intake)*
  All cases emphasized the importance of whom they were with to be able to be (less or more) themselves. For Ella, this was particularly difficult with her mother, who followed a strict religious upbringing.
  *“But I also found out that I’m probably bi(-sexual). Um and also, I think because of my mother, I couldn’t even think about that, because she’s [her mother], every time you see something like that on TV about those things, she was like ‘ah that’s devilish and via TV they want that to get into your head?’ [...] and uh my oldest sister is a lesbian, so my mother was angry about that (hmmm) uh my father is, well he is totally okay with that (hmmm).”*
  (Ella, post-interview)
  For Sam, being acknowledged for who they were was an important theme and recurring struggle, in particular with their father. For instance, they found it crucial for people to respect their non-binary identity by using the correct pronouns. In the intake interview, Sam mentioned that being misgendered felt like a personal attack, so they tried to dress as androgynously as possible hoping to avoid this.
*“With my mom my relationship is very good, we get along very well and talk about everything, she really knows everything about me, I’m non-binary and she’s super accepting about that, and she uses my proper pronouns [unlike my father] and that’s all very good” (Sam, intake).*
  For Kobe, the struggle to be himself mainly occured at home. He experienced a clash between his need for freedom, something he got used to when being away on an exchange for a year, and the limited autonomy he felt being back home with his parents.
  *“ I’m of the opinion that it should be possible, because I’m old enough now and I think that I*
  *should have that freedom, the freedom to do whatever I want every day. And they [parents]*
  *think that I have to consider the people I live with, but to the extreme, and I think that’s where it goes wrong the most, at least at the moment.” (Kobe; post-interview)*
  *A need to share my emotions and be with the “right” people*.Second, all participants expressed a need to share emotions with their parents, partners or friends. This relates to the previous theme that, to be able to be vulnerable and themselves, they needed to be among the right people. All four mentioned the importance of having (the right) friends, colleagues or partner, and even a cat, with whom they could be themselves and share feelings. The importance of being among others, ranged from enjoying time with friends and family (Ella, Sam) to a need to be somewhere else to avoid conflicts (Jan, Kobe).
  “(looking forward to) seeing my girlfriend again and being able to for a moment let go of all the emotions that need to go away and are allowed to go away.” (Jan, ESM morning)
  “When it [conflicts] got really bad, leaving home, for me, mentally that gave me a rest for a while. But then it didn’t go 100% with that family [of his then-girlfriend] anymore and I was like, I have to go back home and have a good conversation. And we had a really good chat and then I decided to stop school and really focus on myself and it was only from then on that things started to get better”. (Jan, post-interview).
  However, all four adolescents experienced that their problems were often not heard or misunderstood, often by (one of) their parents (all cases) or society (Sam).
  “I feel that here at home I don’t get the opportunity to become more mentally stable. Feeling
  bad is not allowed.” (Jan, ESM evening)
  *Learning to prioritize myself through therapy.* Third, the adolescents said they (tried to) prioritize themselves and became more confident about their own choices and needs (e.g., music, talk about emotions, mental peace, education), processes which seemed to have been facilitated in therapy.
  “Um yes um now in the last half year, I’ve really made a lot of changes in my life, positively
  (yes) um. And I’m actually really proud of that, of myself, because I just dare to be selfish.”
  (Ella, post-interview)
  Ella mentioned she had long struggled with her emotional side, as it made her feel different from others. She had mostly kept this side to herself and found it difficult to express her emotions, not wanting pity (intake). After therapy, she started sharing her emotions more; she demanded more attention from her parents and took more time to talk about her sorrows.
  “I’m also a very emotional person. It’s not very practical in this society because… if I… for the
  smallest things, I have to cry, even out of happiness and that’s very exhausting because I don’t
  want that. But actually, it’s okay.” (Ella, post-interview)
  This theme was not central for Sam, who stated that therapy had not been succesful for them and more specialized help was needed. In contrast to prioritizing themself, Sam mentioned a preoccupation with overburdening others with their worries and being annoying for them, which could lead to being abandoned. This was captured by the first theme (difficutly being myself with and due to ohers).
  “But I just think to myself if I open up completely and they know me super well and we are super good friends. I’ll still keep thinking that those people find me annoying and are actually super eager to get away from me.” (Sam, intake).
3. Idiographic networks and subjective experiences: convergence, divergence and complementarity
  Across the four cases, limited convergence was found between their networks and subjective experience, while some divergence was observed where convergence could have been expected. Triangulation mainly pointed at the ways in which the qualitative and quantitative data complemented each other. Considering space, we highlight the most important findings across the four cases.
  *Convergence* First, across cases it was noticed that no dynamics were observed for feeling ashamed, suggesting this node/experience was of less importance or prominence to the youth. Of all cases, Jan’s findings most clearly signaled emotions like sadness, worry and a struggle to be himself in both his network and themes. For instance, Jan’s contemporaneous network (Fig. 3, left), which must be interpreted with caution due to low reliability, showed a positive association (PCC = 0.09) between ‘I feel sad’ (M = 3.69; SD = 1.55) and ‘Just before the notification, I was worrying’ (M = 4.36; SD = 2.00), controlling for other variables in his network: during moments when Jan reported feeling sad, he also indicated experiencing worry. In correspondence, during the post-interview, Jan expressed his past struggles with sudden emotional outbursts and panic attacks on an increasingly daily basis, which felt out of his control. To release these emotions and gain control over them, Jan needed to share them with his friends, his girlfriend, or at school.
  *Divergence* Second, the centrality of concerns about weight and how they looked, voiced by all cases in their interviews and/or open-ended ESM data, was not observed in Jan and Sam’s idiographic networks. This suggests that for them the items ‘being satisfied with looks’ did not fully correspond to the adolescents’ own experiences of “wanting the ideal weight” (Jan). For Ella, the suggested but unreliable predictive association between feeling tired and sad (Fig. 3, right) was contradicted by her qualitative data. In Ella’s experience her “mental fatigue” stemmed from her emotionality, rather than the other way around. While the interviews point at handling emotionality as a possible lead for therapy, the network suggests focusing the intervention on fatigue first.
  *Complementarity* The quantitative ESM data and resulting networks focused the attention to daily fluctuations and dynamics in key symptoms like worrying, sadness and feeling tired. These patterns were not as clearly observed in the open-ended ESM responses and interviews. As the open-ended nature invited participants to elaborate on their experiences, they not always reported on the same topics. This could be because it was simply not on their minds at the time of reporting. Systematically mapping the same items allowed monitoring their presence and intensity over time; for instance, this indicated a dynamic of worrying and sadness for Sam (Fig. 3). Vice versa, the open-ended nature of the qualitative data and resulting themes allowed captured personal meaning and context beyond the pre-set ESM items. For instance, adding information about what Sam may be worried about (e.g., their boyfriend not being there) and generating explanations for why they felt sad (e.g., being misgendered). For all cases, the qualitative data gave insight into their daily lives, the topics that mattered most to them (e.g., being active, Kobe; making music, Ella), and the situations and people that impacted them (e.g., mother, girlfriend). The themes also captured changes resulting from therapy (theme 3), like a change in self-concept for Ella (e.g., finding out I am bisexual), being more aware of what is important (e.g., music). This suggests that the meaning of the item ‘I can be myself’ possibly changed over time.

## Discussion

This study demonstrated the value of a mixed methods approach to enhance the validity of idiographic networks aimed at personalized care by adding meaning and context with qualitative methods. Presenting the results of only four participants, our findings do not allow nor aim at generalization. The in-depth multiple case design and thick description could facilitate transferability of results, however. Triangulation of the quantitative and qualitative results, allowed to examine how they converged, diverged and complemented each other. We end our discussion with suggested ways forward.

First, little convergence was found between the networks and themes, signaling how the quantitative intensive longitudinal (GraphicalVAR) and qualitative (thematic) analyses bring in very different idiographic approaches. Syed (2024) explains how this difference relates to whether idiographic is understood at the analysis and/or inference level. He argues that today’s understanding of idiographic psychology focuses strongly on the level of analysis, i.e., the individual-level instead of the group-level, with intensive longitudinal analyses as concrete example. This however diverges from the original definition by Windelband (1894), who differentiated idiographic from nomothetic on the basis of the inference level, i.e., drawing conclusions about the particular versus the general. A confusion Syed ascribes to Allport (1937), who conflated individuality and idiographic but later (1962) abandoned that alignment. A current side-effect of an analysis-focused interpretation is that idiographic analyses are performed with the nomothetic ideal of generalizability, which has resulted in a dominant use of quantitative methods (Syed, 2024). For instance, intensive longitudinal analyses aiming to identify within- and between-person dynamics in depression and identity. Qualitative research, being idiographic at the level of analysis and inference, does not allow generalization (to the population) but rather aims at in-depth understanding of peoples’ experiences within their own context. For instance, identity experiences of first year LGBTQIA + students in a Dutch university. Across participants, overarching themes can be derived but findings remain context-dependent (i.e., particular). They can be translated to other individuals and contexts based on their similarities with the conditions of the study (e.g., characteristics of the students, the culture of the university), which is called transferability. Thus, while both quantitative and qualitative methods can be used for individual and group-level assessment and analyses, Syed (2024) argues for a definition of idiographic psychology that includes the inference level, as it would pave the way for adopting more qualitative methods. Our study demonstrates the direct added value.

The different definitions of ‘idiographic’ have important consequences for current ESM-based attempts at personalized care, however. Within the first logic, assessing individuals with the same (standardized) items and ESM designs may seem a logical approach to arrive at personalized care. Within the second logic, assessing individuals with the same items and designs works against personalization, as standardization gets in the way of capturing subjective meaning, the nature of peoples’ lives and why certain situations are relevant at which given times. The resulting problem of the first logic was illustrated by how little our networks, based on generic items and designs, could tell us about the four unique ways our cases experienced their identities, their relationships and emotions. Rather than standardization, the qualitative analyses added insights into their subjective experiences and contexts. Crucially, as idiographic assessment does not necessarily imply remaining at the single case or individual level, the term cannot be used interchangeably with personalized assessment (although this is often misleadingly exchanged, see De Smet e.a., 2024 for a discussion). Hence, idiographic methods, quantitative or qualitative, mean a valuable but not a sole or final step towards personalized care. This need has been echoed by calls for actual personalized assessment (e.g., Sales, 2017).

Second, the qualitative data signaled diverging results that helped cross-validate observed associations and provided possible alternative explanations. Our findings warn against interpreting the directed associations in the network as simple causal relationships (see case Ella). Indeed, VAR-models present predictive relationships over time, not causality. Moreover, conclusions are limited to the nodes that were included in the network analyses, which are limited to only a few variables (typically with a maximum of six). In other words, it cannot speak about dynamics that were not included in the network. Hence, they are a poor means to capture actual causal mechanisms (Bringmann et al., 2024; Oude Maatman & Eronen, 2025).

Third, the quantitative ESM data allowed for the systematic monitoring of specific symptoms across time, while the qualitative data captured domains and processes that were not covered by the nodes and edged. As said, the pre-defined and generic ESM items (selected from existing items in the repository) did not capture the subjective meanings of the different cases. Beyond abstract constructs like worrying and sadness, case-specific concerns were captured in adolescents’ own words, like experiencing “mental fatigue” (Ella). In addition, the open-ended ESM and interviews also proved complementary. Notably, the interviews captured relevant therapeutic changes, like a change in self-concept (e.g., finding out I am bisexual), which could not be identified from the quantitative data alone. Repeated measurement of the same item assumes stable meaning within participants over time, yet open-ended ESM items can help assess response shifts more directly (see Schorrlepp & Maciejweski, 2024).

The VAR-based networks served as an illustration of current endeavors to establish idiographic networks for personalized care; several inherent limitations are important to consider for our findings and further applications (see Bringmann et al., 2024). In particular, the assumption of time-invariance in VAR-models is problematic to capture changes facilitated in psychotherapy as observed in our cases. Non-stationary networks are a more suitable alternative (for relevant examples see Hulsmans et al., 2024a, Olthof et al., 2023, and Schiepek et al., 2016).

Critically, idiographic networks (VAR-based and others) are as robust as the quantity and quality of the data collected. More observations enhance the robustness and interpretability of the networks. However, even if networks perform as optimal as intended, on their own they cannot capture the story of the patient. This subjective understanding (e.g., what “being myself” means to a patient) is the ultimate added value of qualitative data and as such indispensable for personalization in clinical care.

### Validation Through Triangulation: Four Reasons Why

Based on our multiple case study, we outline four (non-exhaustive) reasons for using triangulation to enhance the validity of idiographic networks based on ESM data.

### Reason 1: 1 + 1 Means 3, a Confirming and Complementary Picture

First, the oldest and most frequently used understanding of methodological triangulation is the aim of verification (Denzin, 2012). However, this narrow focus only considers what is shared, and as such potentially neglects the wealth of information gained by combining different methods. Therefore, more informative, and doing right to studying complex human phenomena using different methods, is to investigate not only what they share, but also what they uniquely add. Mixed methods can thus identify what is covered by the ESM-based idiographic network and what is added by the qualitative strand, and vice versa. In our study, convergence was limited, but all four cases illustrated insightful complementarity.

### Reason 2: Numbers and Narratives Tell a Different Story, a Contrasting Puzzle

Second, the logical counterpart of the first reason is diverging or contrasting results. Divergence can enhance the validity in two powerful ways. First, results from one method can be used as the reference point or norm to cross-validate the findings of the other. For instance, when using patients’ subjective experiences to assess item validity (see De Smet, 2024). Second, divergence presents us with a Rashomon effect^1^: contrasting views generate several challenging hypotheses, where it is unclear which one is (most) correct. Rather, this puzzling observation can instigate novel angles for further investigation into several potential explanations. In our study, this scenario was observed for Ella.

### Reason 3: Items Stay the Same, But Their Meaning Changes Over Time

A third reason to add qualitative data to repeated measurement of patients/participants, is that the interpretation of what items mean can change over time; a phenomenon more generally known as response shift (Schwartz et al., 2004). As this cannot be deciphered from the quantitative data, the qualitative strand adds crucial meaning to interpret results, in effect, changing the meaning of the ESM data and resulting networks. While this can flag important validity issues for the statistical interpretation of the networks, at the same time it adds rich contextual information relevant for clinical conclusions regarding change over time. In our study, this was observed for several cases as an effect of therapy.

### Reason 4: (Too) Many Numbers are Missing But There is a Rich Story Behind it

A fourth reason, and common situation when collecting intensive longitudinal data, is dealing with missing observations. A common research practice is to omit these cases given they have insufficient data to perform statistical analyses. However, missing data can hold significant clinical information. When unrightfully treated as missing at random, this threatens the validity of conclusions. Qualitative data can provide extra context that may explain missingness and/or allows to make meaning of even limited data points. In our study, this was found for Kobe, a case we deliberately selected because of his rich descriptions of identity experiences yet remarkably limited ESM data, despite his self-declared motivation to participate in the study.

### Strengths, Limitations and Future Directions

This study demonstrates the value of a mixed methods approach to assess and enhance the validity of idiographic networks by adding meaning and context to nodes and edges using qualitative methods (Bringmann, 2021; De Smet et al., 2024). For example, it became clear why participants could (not) be themselves or worried (e.g., being around the right people; conflicts at home). This is crucial information for personalized care that is not captured by purely quantitative monitoring of generic symptoms. However, the potential of qualitative data-collection with ESM could be further expanded. The number of open-ended questions and responses were still limited in our study. Open-ended ESM items too are vulnerable for missingness, and careful instruction and respondents’ burden should be considered. Relying on different sources of qualitative data (e.g., open-ended ESM and interviews) and using a wider range of items and response modes, tailored to patient’s preferences, is recommended (e.g., text, voice, video, photo).

The employed mixed methods parallel design allowed triangulation of quantitative and qualitative results, ideal for validity assessment and complementary perspectives. However, as both strands were collected separately, the same pre-defined ESM items and assessment schedules were therefore applied to all cases, (wrongly) assuming similar relevance, item interpretations and daily rhythms. As such, we may have missed important observations and fluctuations due to assessing the wrong variables at the wrong time. Indeed, the resulting networks depend entirely on the pre-selected variables and chosen lag-intervals, which are often unknown and therefore chosen for pragmatic reasons (Epskamp et al., 2018a).

### Enhancing Personalization Using Sequential Mixed Methods Designs

The power of mixed methods idiographic networks could be further enhanced by using sequential designs, in which one strand builds onto the other (Hesse-Biber, 2010). Working exploratory (qual — > quan), personalized items and optimal assessment schedules could then be developed a priori, for instance during an intake interview, minimizing the risk of irrelevant or missing data. Working explanatory (quan — > qual), a follow-up interview could discuss observed patterns or networks in more depth. Ideally, in multiple waves, data-collection and analyses could be continuously improved (e.g., adding items, capturing changing interpretations, adjusting items). This would iteratively increase the validity of the collected data and constructed idiographic network, by capturing meaning and context from the start. For an example of such approach in clinical practice see Schiepek et al. (2016).

Hence, to move idiographic networks forward in their ambition for personalized care, it is a priority to optimize ESM designs to ensure personalization from the onset. Thoughtful conceptual work should specify the constructs and dynamics we wish to observe (Bringmann et al., 2022b; 2024; De Smet et al., 2024), and grasping patients’ personal story should be at the forefront of personalized assessment and care. Qualitative methods offer a powerful means here, closely tied to actual clinical practice.

## Electronic Supplementary Material

Below is the link to the electronic supplementary material.

Supplementary Material

## Footnotes

1 The effect is named after Akira Kurosawa's 1950 film Rashomon, in which a murder is described in four contradictory ways by four witnesses. The term addresses the various perspectives and points of view that can be taken on a single event. It is used as a metaphor for an epistemological stance that welcomes multiple perspectives to study complex reality.
