# Supplementary material for: Adding Meaning and Context to Nodes and Edges: A Mixed Methods Approach to Enhance the Validity of Idiographic Networks
Source: Cognit Ther Res. 2025 Nov 10;50(4):907–21. doi: 10.1007/s10608-025-10670-6 (PMC13469479; doi:10.1007/s10608-025-10670-6)
Supplement: Supplementary file 1 [file 10608_2025_10670_MOESM1_ESM.docx]

**Appendix methods**

**Preregistration and Open Science**

The study was not pre-registered. All materials, anonymized ESM data and R scripts can be found on OSF ([https://osf.io/jgz7t/)](https://osf.io/jgz7t/). Given the sensitivity of the qualitative data, they are not openly available. Additional information can be obtained via the corresponding author upon reasonable request.

**Case Vignettes**

**Case I. Jan** (he/his) sought therapy due to experiencing significant school-related stress, resulting in quitting university during the period of the study. He encountered many intense conflicts with his mother, which resulted in a period of moving away from home. Jan had been bullied for several years; like his brother, he was being picked on because of his weight. This has stimulated (extreme) dieting and doing (excessive) sports. At the end of therapy he decided to study to become a nutrition specialist. He broke up with his girlfriend over the course of the study.

**Case II. Ella** (she/her) sought therapy for what she called “her persistent feelings of loneliness” and suicidal ideations. She grew up in a devoutly religious household, and particularly her mother held strong religious convictions. She has two siblings; one sister suffers from mental health issues. Ella worried a lot about her sister, for instance regarding her non-compliance to medication. During the covid pandemic, Ella’s father was admitted to the hospital in life threatening conditions and had to remain there for a couple of weeks. This (uncertain) period left a huge impression on Ella and the bond with her father grew stronger.

**Case III. Sam** (they/them) sought therapy for OCD complaints, which they attributed to the experience of being bullied and excluded by peers. Since the age of ten, Sam had engaged in several therapeutic interventions. Their parents are divorced. At the time of the study, Sam was living with their mother, and no longer wanted contact with their father. Sam’s brother had chosen the side of their father, which caused tension between the siblings. At intake, Sam discussed considering chest surgeries and doubted which pronouns fitted best. In the post-interview, Sam expressed being certain about their bisexuality and non-binarity.

**Case IV. Kobe** (he/his) sought therapy because he felt disconnected from his peers and felt highly insecure about relationships. During a one-year sabbatical after high school he had spent six months abroad for an exchange program. After his return, he could no longer connect with his friends. Unlike him, they had already started university and were immersed in their new lives. Two of his closest friends had cut off all communication without explanation, leaving Kobe with doubt about himself and distrusting friendships. After being on his own feet for six months, he struggled with living back at home with his parents.

**Model fit.** The fit of the graphical network models was examined using a latent network model (LNM), following the methodology outlined by Epskamps e.a. (2018a) and implemented in the lvnet package (version 0.3.5) in R. We evaluated model fit using the RMSEA, where values between 0.08 and 0.06 indicated an acceptable fit and values < 0.06 indicated a good fit (Browne & Cudeck, 1993). The reliability of the idiographic networks was evaluated using the split-half method, following the methodology outlined by Beck and Jackson (2020). The responses of each participant were divided into two sets: a mid-split and an odd-even split, accounting for the passage of days. The mid-split approach categorized data into the first half versus the second half of the days, while the odd-even split segregated data based on even versus odd days. For each split (mid and odd versus even), we constructed idiographic temporal and contemporaneous networks for each participant and calculated the correlation between the estimated coefficients across the two halves as a measure of reliability (Beck and Jackson, 2020). This resulted into two temporal and two contemporaneous networks for each participant for each type of split; eight per participant in total. We next assessed the stability of the idiographic networks across pairs of contexts, using the split-half method (Beck & Jackson, 2020). The chosen context splits are related to either specific social company or location. For instance, a location context split could be home vs not home; a social company context split could be with partner vs not with partner. These splits were different for each participant (see Table 3-5), informed by the qualitative analyses (e.g., significance of a partner mentioned in the interviews) and the frequency of a reported context in the quantitative ESM data (e.g., being home). Although the same methodology was used (Beck & Jackson, 2020) the resulting correlations were interpreted as measures of network stability across context, rather than as measures of reliability. Due to the limited data points across these contexts, it was not possible to estimate idiographic temporal and contemporaneous networks for all contexts, resulting in only limited correlations across contexts. Interested readers are referred to OSF for the R codes for these analyses (<https://osf.io/jgz7t/>).

Finally, the underlying assumptions of the model have important implications for assessing and interpreting the reliability of dynamic networks. Under the assumptions of the presented VAR model, i.e., time-invariant associations, stable networks over time are a sign of reliability. In this study, the split-half reliability was low for both contemporaneous networks and temporal networks; in one case there was too little data to estimate the split half reliability. While we relied on the approach by Beck and Jackson (2020), Hoekstra e.a. (2024) propose an alternative approach for testing reliability of idiographic networks, however, this requires a high number of datapoints (t >100) per individual, which is often not feasible in clinical or research contexts. A potential implication of the low reliability of the ESM measurements in the VAR-based networks is that it may result in missing and spurious connections (Epskamp, Borsboom & Fried, 2018b). However, under the assumptions of the presented VAR model, i.e., time-invariant associations, stable networks over time are a sign of reliability. Yet, when associations are assumed to be time-variant and networks are expected to change, the instability of the network could signify clinically meaningful change. Particularly over the course of therapy, change in networks is hoped for, stimulating the use of less conservative models to capture idiographic networks. Assessing the reliability of idiographic networks is an ongoing challenge (Bringmann, 2021; Schuurman & Hamaker, 2019).

**Appendix results**

Only for one of the four cases, Sam, the resulting networks could be reliably interpreted., hence these networks should be interpreted with caution. Figure 1 presents a summary of all participants’ networks. The observed fluctuations per item over time for each case can be found in Table 1 below. Table 2 presents all themes for each case, separate for the ESM and interviews.

**Figure 1.**

Partial Contemporaneous and Directed Correlation Networks per Participant

|  |  |
| --- | --- |
|  |  |
|  |  |
|  |  |

*Note. Partial contemporaneous correlation networks (left); partial directed correlation networks (right). Circles (nodes) represent variables of identity, self-esteem and symptoms of depression. Green edges indicate positive relationships, red edges indicate negative relationships. The thickness and saturation of the connections indicates the strength (absolute value) of the relationship.*

**Table 1** *Overview of Fluctuations in the Seven Items over 20 days per Participant*

| Jan   | Ella   |
| --- | --- |
| Sam   | **Kobe**   |

**Table 2***.*

Summary of Themes from the ESM Diary and Interviews per participant

| **Case** | **Case-specific themes** | |
| --- | --- | --- |
| **Jan** | ESM diary | The importance of being among people, away from conflicts at home  The importance of being able to let go of my emotions  The importance of finding mental peace |
|  | Interviews | Self-esteem depending on school stress and weight loss  Strong sense of justice for myself and others  Need to share my emotions with others  From doubting my own choices to choosing for myself and my joy |
| **Ella** | ESM diary | Wishing to talk and be in (good) company, like friends  Coping with “mental fatigue” alone while parents do not understand  The importance of making music |
|  | Interviews | Grateful to be with my father and friends (good company)  Balancing doing something for others versus myself  Music is me  From handling “mental fatigue” alone to sharing my emotional side  Being myself as bisexual and non-religious, except with my mother |
| **Sam** | ESM diary | The importance of being with my partner and cat  Daily complaints: feeling anxious, stressed, depressed or having problems with my weight |
|  | Interviews | Longing recognition for Neurodivergence, Non-Binarity and Bisexuality  The importance of having my mother and partner close  Fear of annoying others and be abandoned |
| **Kobe** | ESM diary | Feeling good when I am productive by doing activities outside, with friends and feel in control over my daytime activities  Having recurring arguments with my girlfriend and parents |
|  | Interviews | Unlocked maturity but having no autonomy at home  Slowly breaking out of the vicious cycle of self-doubt  The importance of looking fit  Need to share my problems with others |

**Case I: Jan.**

**Jan’s idiographic network.** Figure 1 (a,b) shows Jan’s networks^[1]^.  Conditioning on the lagged effects, a thin unsaturated green edge was observed between ‘I feel sad’ (*M* = 3.7; *SD* = 1.6) and ‘Just before the notification, I was worrying’ (*M* = 4.4; *SD* = 2.0), indicating a positive association (PCC = .09). This suggest that during moments when Jan reported feeling sad, he also indicated to worry. A red saturated edge was observed between ‘Just before the notification, I was worrying’ and ‘I can be myself’ (*M* = 3.6; *SD* = 1.3), controlling for other variables in his network (Figure 1, a). This indicates a negative association (PCC = -.17), which suggests that reporting increased levels of worrying, coincided with reporting a decreased ability to be himself. Conversely, reduced levels of worrying, coincided with a higher level of being himself. A thin unsaturated red edge was observed between ‘I can be myself’ and ‘I feel sad’, indicating a negative association (PCC = -.07), controlling for other variables in his network. This suggests that each time he reported a diminished ability to be himself, he also indicated an increased feeling of sadness at the same measurement point, and vice versa. Finally, no associations were observed in Jan’s temporal network (Figure 1, b).

**Central themes in Jan’s ESM diary.** The central themes reported in Jan’s diary were *the* *importance of being among people, away from conflicts at home, being able to let go of my emotions* and *finding mental peace.* Being at home was often reported as the most negative event due to arguments with his parents and brother; being with his girlfriend, colleagues or at the university meant a moment of rest (most positive event of the day).

*"I feel that here at home I don't get the opportunity to become more mentally stable. Feeling bad is not allowed." (evening)*

*"(looking forward to) seeing my girlfriend again and being able to for a moment let go of all the emotions that need to go away and are allowed to go away." (morning)*

Jan often reported on experiencing stress, eating or talking about food, multiple times a day. Cooking was reported as a positive event. When Jan could “wake up peacefully” or could be “at peace mentally”, he reported this as a positive event.

**Central themes in Jan’s interviews at intake and post therapy.**

***Self-image depending on school stress and weight loss.*** School gave Jan more energy, perseverance, and motivation. However, Jan quit university during the exam period as his stress levels had become too high to deal with (post-interview). He noticed how stress impacted his self-esteem (intake).

*“My self-esteem was better again, and motivation to do fun things again, and to get myself through it, and now with more stress I noticed that it has been pulled down again, that I needed to come here [therapy] again and tell my story again” (intake)*

Jan was highly concerned with how others perceived him, which made him put a lot of focus on losing weight. However, although he looked different physically, he still perceived himself (negatively) the same as before; while looking more “fit”, he felt worse mentally.

*"My brother crashed... Without me realizing it, he had started a sort of crash diet, and I thought, if he can do it, so can I. I lost a lot of weight, which made me feel better at the time, but mentally worse and worse." (intake)*

***Strong sense of justice for myself and others.*** Jan described having a strong sense of justice, which meant respecting others and doing everything as correctly as possible. It was challenging to interact with classmates who did not share those values. Also in discussions with his mother, his values clashed heavily with her opinions (intake).

***Need to share my emotions with others.*** Jan said he struggled with sudden emotional outbursts and panic attacks. To release these emotions and gain control over them, Jan needed to share them with his friends, his then-girlfriend, or at school. After therapy, he sought more social contacts, whereas previously he tended to block them out (intake). At home, Jan felt his feelings were not understood, resulting in arguments about his mental well-being with his parents (intake). He said his parents tended to normalize what he felt, explaining it as part of “his puberty” (intake). A temporary move to his then-girlfriend made it possible to finally talk to his parents about his need to share his emotions. Since then, his relationship with his parents and siblings improved (post-interview).

*“When it got really bad, to leave home, for me mentally that gave me a rest for a while. But then it didn't go 100% with that family [of his then-girlfriend] anymore and I was like, I have to go back home and have a good conversation. And we had a really good chat and then I decided to stop school and really focus on myself and it was only from then on that things started to get better”. (post-interview)*

***From doubting my own choices to choosing for myself and my joy.*** Jan learned to prioritize his own well-being, such as focusing on things he enjoys (e.g., studying nutrition). Before therapy this was not the case, Jan mentioned in both interviews. He used to let others make choices for him and would sometimes support others at his own expense. In the intake interview Jan explained he always doubted the choices he made and struggled to accept them, contemplating that an alternative choice might have made him feel better.

*"I can fully focus on what I like to do, without feeling bad about making that choice for myself." (post-interview)*

**Case II. Ella**

**Ella’s idiographic network.** Figure 1 (c, d) displays the two networks for Ella^[2]^. A thick red edge between ‘I can be myself’ (*M* = 5.6; *SD* = 1.1) and ‘Just before the notification, I was worrying’ (*M* = 3.5; *SD* = 2.1) (see Figure 1, c) represents a negative association (PCC = -.08) controlling for other variables in her network. This suggests that when Ella reported increased worrying, this coincided with a decreased ability to be herself at the same measurement point. Vice versa, this suggests that when Ella worried less, she could be more herself at that moment. Furthermore, a thin green edge was observed between ‘Just before the notification, I was worrying’ and ‘I am tired’ (*M* = 4.5; *SD* = 1.7), which represents a positive association (PCC = .04) controlling for other variables in her network. This suggests that each time Ella reported that she was worrying, she also indicated feeling more tired, and vice versa. Additionally, a thick green saturated arrow between ‘I am tired’ and ‘I feel sad’ (*M* = 5.5; *SD* = 1.9) was observed in her temporal network (Figure 1, d), meaning that at one measurement point ‘I am tired’ seemed to predict ‘I feel sad’ in the next’ (PDC = .16). This suggests that when Ella reported increased fatigue, she expressed feeling sadder at the subsequent measurement point. Furthermore, a thin unsaturated green arrow was observed (Figure 1, d) for ‘I feel sad’, indicating a positive autoregression (PDC = .06). This suggests that when Ella reported being sad, she reported being sadder at the subsequent measurement point. Finally, a thick green arrow was observed for ‘I am satisfied with how I look’, indicating a positive autoregression (PDC = .16). This suggests that when Ella felt satisfied with her looks, the next measurement point she felt more satisfied with her looks.

**Central themes Ella’s ESM diary.** The main themes in Ella’s diary were *wishing to talk to and be in (good) company* and *coping with “mental fatigue” alone while parents do not understand*. Being able to talk or be with co-workers, friends, her therapist or her sister was reported as important to her. *Making music* returned as a central important daily activity.

*"Whenever someone gave me hope to do something with me I became happy but I was rejected more than once because my friends had to do something else so now I'm just more gloomy." (evening)*

Talking to her mother was reported as a highly negative event. She for instance expressed hopes her mother would “learn to listen better”. When it came to dealing with her emotions or negative events, Ella did not report on turning to friends or family. Ella indicated several times that she had to weep or sleep when a negative event had occurred.

*"My dad flipped on me today and at that point I wanted to run away but I had a good cry and then suddenly I fell asleep around 3pm and I think that’s from being mentally tired, but after that I felt a little better.” (evening)*

**Central themes in Ella’s intake and post therapy interviews.**

***Grateful to be with my father and friends (good company).*** Ella mentioned the importance of being in good company, especially with her father (which intensified after his critical hospitalization), with whom she shares music taste and humor. In contrast, she expressed having a conflicted relationship with her mother.

*"I really appreciate every moment that he (her father) is with me, even if he's angry, even if there-, if we don't have a nice moment together. I'm just happy that everyone exists, that everyone is healthy, that we can be euhm...yes...outside every day. and that actually sounds so very dramatic but I'm really genuinely happy about that..that I can see my friends every day." (post-interview)*

***Balancing doing something for others versus myself.*** Ella explained she struggled to find a balance between considering her own needs and those of others, as she found joy in the happiness of others. In the post-interview, she mentioned that in the past, she did not think about herself enough. She felt proud that she dared to be more selfish by doing things she enjoys and expressing her opinions.

*"I also just realized because of corona [covid-19 pandemic] that I have to take care of myself more and should not always do everything for others, because otherwise, I will remain sad, but I also don't have to only think about myself, because then others will [be sad], so there has to be a balance, and I'm trying to find that now." (intake)*

*"Um yes um now in the last half year, I've really made a lot of changes in my life, positively (yes) um. And I'm actually really proud of that, of myself, because I just dare to be selfish." (post-interview)*

***Music is me.*** Making music (i.e., playing an instrument; studying music) had a central place in Ella’s life. This gave her the opportunity to do something for herself while moving others. In her post-interview she explained to have found more joy in playing music.

*"Music is so emotional, and it actually makes me very happy that I can play it every day, listen to it, and study it, and that's also a kind of therapy for me. So, and I believe for a lot of people in this world. And that really puts me at ease, that I contribute something to the world." (post-interview)*

***From handling “mental fatigue” alone to sharing my emotional side.*** Ella mentioned she has long struggled with her emotional side, as it made her feel different from others. She had mostly kept this side to herself and found it difficult to express her emotions, not wanting pity (intake). After therapy, she started sharing her emotions more; she demanded more attention from her parents and took more time to talk about her sorrows.

*"I'm also a very emotional person. It's not very practical in this society because... if I... for the smallest things, I have to cry, even out of happiness and that's very exhausting because I don't want that. But actually, it's okay." (post-interview)*

Moreover, Ella saw her emotionality as a reason for her “mental fatigue”, draining her energy.

***Being myself as bisexual and non-religious, except with my mother.*** Ella explained that she used to hate herself and felt ugly. By caring less about what others thought of her, she could do and wear as she pleased. She identified as bisexual, which she did not talk about at intake, suggesting the topic emerged throughout the therapy process.

*“But I also found out that I'm probably bi. Um and also, I think because of my mother, I couldn't even think about that, because she's [her mother], every time you see something like that on TV about those things, she was like 'ah that's devilish and via TV they want that to get into your head?' [...] and uh my oldest sister is a lesbian, so my mother was angry about that (hmmm) uh my father is, well he is totally okay with that (hmmm).” (post-interview)*

She had also found out she does not believe in God. However, only with her mother she still “couldn't fully be herself” (post-interview).

*“I was able to say to him [her father] a while ago, ‘I don't think I believe in God, but I don't think there is anything, but I don't know that and I don't actually want to know’ and we could just talk about that. And my mother, I still don't dare say that, I think she would, yes, be super angry and super sad. So yeah, I didn't have that space to say that yet.” (post-interview)*

**Case III. Sam**

**Sam’s idiographic network.** Figure 1 (e, f) shows the two networks for Sam^[3]^. Figure 1, e visualizes a thick green edge between ‘I feel sad’ (*M* = 2.4; *SD* = 0.9) and ‘Just before the notification, I was worrying’ (*M* = 3.4; *SD* = 1.3), indicating a positive association (PCC= .08) controlling for other variables in her network. This indicates that whenever Sam reported to feel sad, they concurrently reported to worry. Figure 1.f indicates a thick green arrow for ‘I feel sad’, indicating a positive autoregression (PDC = .20). This shows that when Sam reported feeling sad, they felt sadder at the next measurement point.

**Central themes in Sam’s ESM diary.** The open-ended responses in Sam’s diary were generally more limited in number than for the other cases. Nonetheless two main themes in Sam’s ESM diary were identified: the *importance of being with my partner and cat*, and *daily complaints:* *feeling anxious, stressed, depressed or having problems with my weight.* Sam recorded being with their partner 22 times as a positive and significant event. When they did not have much time with their partner, it was noted as a negative event. Sam reported feeling anxious on several occasions, such as when their partner changed their plans.

*"My partner made the day better." (evening)*

*"My partner let me know that they will have to work all Summer, so we won't be able to do a lot together." (evening)*

*"I also had more problems today with my weight and feeling fat. This has started again since I weighed myself last week." (evening)*

**Central themes in Sam’s intake and post therapy interviews.**

***Longing recognition for Neurodivergence, Non-Binarity and Bisexuality****.* Sam deliberately said they did not want to be seen as neurotypical, since their multiple diagnoses entailed various difficulties, such as being easily overwhelmed and overstimulated. In both the intake- and post-interview, the importance of receiving diagnoses was emphasized as it helped Sam better understand themself. However, others did not acknowledge Sam’s diagnoses, approaching Sam as neurotypical, minimizing the paired difficulties and perceiving it as 'special'. Until others adequately acknowledge the severity of their diagnoses and neurodivergence, Sam prefers it to be recognized as having a disability. Additionally, their non-binary identity and bisexuality were significant components of their identity. Sam found it crucial for people to respect their non-binary identity by using the correct pronouns. In the intake interview, Sam mentioned that being misgendered felt like a personal attack, so they tried to dress as androgynously as possible hoping to avoid this. For instance, Sam’s father doesn’t make an effort to use the right pronouns (intake).

*"And those are actually the things that shape my personality the most: I have ADHD, I am autistic, and I have OCD. These are the things that make me me. The fact that I am very energetic but also have a lot of difficulty with many social things, those are two contradictory things sometimes, but that's because I have ADHD and autism at the same time." (intake)*

***The importance of having my mother and partner close.*** In the intake interview, Sam explained that they can talk about their problems with their mother and partner, which helps them put things into perspective.

*“With my mom my relationship is very good, we get along very well and talk about everything, she really knows everything about me, I'm non-binary and she's super accepting about that, and she uses my proper pronouns [unlike my father] and that's all very good” (intake)*

***Fear of annoying others and being abandoned***. In the post-interview Sam disclosed that all their problems and fear of abandonment stem from the fear of being perceived as annoying, due to bullying experiences in the last three years of high school. Their classmates were “sweet in their face but laughed at them behind their back”. Sam mentioned that their insecurity hindered them from opening up to new friendships.

*“But I just think to myself if I open up completely and they know me super well and we are super good friends. I’ll still keep thinking that those people find me annoying and are actually super eager to get away from me." (intake)*

**Case IV: Kobe**.

**Kobe’s idiographic network**. Figure 1 (g,h) shows the two networks for Kobe^[4]^. Controlling for the other variables, a thick green saturated edge between ‘I am satisfied with how I look’ (*M* = 3.4; *SD* = 1.9) and ‘I like myself’ (*M* = 3.0; *SD* = 0.9), indicate a positive association (PCC = .03) (Figure 1.g). This suggests that moments when Kobe liked himself corresponded to him reporting greater satisfaction with his appearance. In addition, in Kobe’s temporal network (Figure 1, h) a smaller saturated red arrow between ‘I feel sad’ (*M* = 2.2; *SD* = 0.6) and ‘I was worrying’ (*M* = 2.3; *SD* =1.0) was observed. This indicates a negative predictive association (PDC = -.17) corrected for the negative autocorrelation of ‘Right before the notification, I was worrying’ (PDC= -.04). This autocorrelation suggests that when Kobe was worried at one time point, he worried less at the next. Additionally, this suggests that moments when Kobe reported feeling sad were followed by reporting less worrying next.

**Central themes in Kobe’s ESM diary.** Central in Kobe’s diary were *feeling good when I am productive by doing activities outside, with friends and feel in control over my daytime activities,* and *having* *recurring* *arguments with my girlfriend and parents.* For Kobe, being productive and active preferably meant being outdoors and with friends. Positive events in his diary were for example, biking to the city, painting, cooking food or going for a swim with his girlfriend, reading, visiting his grandmother. He wrote: *"I realize that being productive does me good,"* If he failed to do something outdoors, it was a "wasted" day. Key was the feeling of having control over this, which was often limited

*"I really want to do things outside, but for stupid reasons, that often doesn't happen. I feel like even though those reasons are within my control, I still don't have complete control over them because I live with others." (evening)*

He would often reflect on arguments he had had, especially with his girlfriend.

*"I’ve been unrightfully angry with my girlfriend, now I feel guilty about it because I love her." (evening)*

**Central themes in Kobe’s intake and post therapy interviews.**

***Unlocked maturity but having no autonomy at home.*** During the six months he spent abroad, he had to manage on his own, not speaking the language, not knowing anyone. At first he had felt very lonely, but afterwards he felt wiser and more mature. Since he came back, he had a strong desire for autonomy, yet he struggled living at home again.

*" I'm of the opinion that it should be possible, because I'm old enough now and I think that I should have that freedom, the freedom to do whatever I want every day. And they [parents] think that I have to consider the people I live with, but to the extreme, and I think that's where it goes wrong the most, at least at the moment.” (post-interview)*

***Slowly breaking out of the vicious cycle of self-doubt.*** Kobe identified his low self-esteem as one of his major issues for therapy in the intake interview, which manifested in doubts about himself (e.g., whether he was a good person) and the choices he had made in the past. He described being trapped in a vicious cycle: when feeling bad he felt his self-esteem lower, yet his low self-esteem made him feel bad, even at random moments (intake).

*"And that doubt is connected to that low self-esteem and (hmmm). So that's also a very vicious circle actually. Because one enforces the other and vice versa." (intake)*

After therapy, Kobe felt better in his skin and less insecure. Some days he even felt proud of himself, but saying something positive about himself remained unnatural (post-interview).

*"Just being able to honestly say, 'Yeah, I think I'm cool, and I think I have a cool character. And I think I'm intelligent’ or something like that, I would really like to have the confidence to say that (hmm) but I can't and I can't take myself seriously when I say those things." (post-interview)*

***The importance of looking fit.*** Kobe’s physical appearance played a major role in his self-esteem, ever since his mother commented once that he had gained weight. He started focusing on looking good and seeking affirmation for his looks from others. He felt guilty after eating a meal yet at the same time he loved to eat; he also blamed himself for being unhappy with his looks, because he had been exercising less (post-interview).

*“Because I'm also, I'm, I'm also very critical of my looks. And I think that's extremely, extremely important that, that I look good and that people also think I look good and stuff. And since I've been working even harder on that as in, that it's not just my clothes or my face anymore but also the rest of my body, um, I have the feeling that that's really starting to become so unhealthy for myself, ma (hmmm) yeah how do you say that, that's really such a th...that's such a thing in your head, that's really a voice in your head, you can't do anything about it.” (post-interview)*

***Need to share my problems with others.*** At intake, Kobe expressed the need to share his concerns with others. However, with his former group of friends (who had cut him off), his girlfriend and his parents, he felt there was no space to discuss such heavy topics; feelings were never shared. This made him sometimes angry. He only had a few new friends with whom he could talk openly about problems.

*“And so the fact that he [friend] shows that [his feelings] too, does invite me to show mine too, so I do. Yes (hmmm) so with him yes, but with others not really. With my parents...definitely not. And with my girlfriend not really either.” (post-interview)*

**Table 3**

*Profile Correlations from the Split-half Analysis per Context, per Participant*

| PPID | Type | Split_type | Context_var | r |
| --- | --- | --- | --- | --- |
| Jan | Contemporaneous | context | loc_3vsrest | -0.018 |
| Jan | Contemporaneous | context | soc_2vsrest | NA |
| Jan | Temporal | context | loc_3vsrest | 0.238 |
| Jan | Temporal | context | soc_2vsrest | NA |
| Ella | Contemporaneous | context | loc_3vsrest | 0.248 |
| Ella | Contemporaneous | context | soc_1vsrest | NA |
| Ella | Contemporaneous | context | soc_4vsrest | 0.146 |
| Ella | Temporal | context | loc_3vsrest | -0.058 |
| Ella | Temporal | context | soc_1vsrest | NA |
| Ella | Temporal | context | soc_4vsrest | 0.064 |
| Sam | Contemporaneous | context | loc_34vsrest | NA |
| Sam | Contemporaneous | context | soc_1vsrest | NA |
| Sam | Contemporaneous | context | soc_2vsrest | NA |
| Sam | Temporal | context | loc_34vsrest | NA |
| Sam | Temporal | context | soc_1vsrest | NA |
| Sam | Temporal | context | soc_2vsrest | NA |

**Table 4**

*Context Splits from the Split-half Analysis per Context per Participant*

|  | Location | Social company specific |
| --- | --- | --- |
| Jan | Home/room own (=3) vs not home (rest) | Partner (=1) vs rest  Housemates (=2) vs rest |
| Ella | Home/room own (=3) vs not home (rest) | Partner (=1) vs rest  Friends (=4) vs rest |
| Sam | Home/room own (=3) and home/room other (=4) vs not home (rest) | Partner (=1) vs rest  Housemates (=2) vs rest |
| Kobe | Home/room own (=3) and home/room other (=4) vs not home (rest) | Partner (=1) vs rest |

**Table 5**

*Visual Representation Context Splits per Participant*

**Jan: loc_3vsrest**

**Ella : loc_3vsrest**

**Ella - soc_4vsrest**

**References**

Beck, E. D., & Jackson, J. J. (2020). Consistency and change in idiographic personality: A longitudinal ESM network study. *Journal Of Personality And Social Psychology*, *118*(5), 1080–1100. <https://doi.org/10.1037/pspp0000249>

Bringmann, L. F. (2021). Person-specific networks in psychopathology: Past, present, and future. *Current Opinion in Psychology, 41*, 59–64.https://doi.org/10.1016/j.copsyc.2021.03.004

Browne, M. W., & Cudeck, R. (1992). Alternative Ways of Assessing Model Fit. *Sociological Methods & Research*, *21*(2), 230–258. https://doi.org/10.1177/0049124192021002005

Epskamp, S., Van Borkulo, C. D., Van Der Veen, D. C., Servaas, M. N., Isvoranu, A., Riese, H., & Cramer, A. O. J. (2018a). Personalized Network Modeling in Psychopathology: The Importance of Contemporaneous and Temporal Connections. *Clinical psychological science, 6(3),* 416–427. https://doi.org/10.1177/2167702617744325

Epskamp, S., Borsboom, D. & Fried, E.I. (2018b). Estimating psychological networks and their accuracy: A tutorial paper. *Behavior Research Methods 50(1)*, 195–212. https://doi.org/10.3758/s13428-017-0862-

Hoekstra, R. H. A., Epskamp, S., Nierenberg, A. A., Borsboom, D., & McNally, R. J. (2024). Testing similarity in longitudinal networks: The Individual Network Invariance Test. *Psychological methods*, 10.1037/met0000638. Advance online publication. https://doi.org/10.1037/met0000638

Schuurman, N. K., & Hamaker, E. L. (2019). Measurement error and person-specific reliability in multilevel autoregressive modeling. *Psychological Methods*, *24*(1), 70–91.<https://doi.org/10.1037/met0000188>

1. The model fitted the lag-0 and lag-1 covariances acceptable, χ2(48) = 61.16, p = .1, root mean square error of approximation (RMSEA) = .07, 95%, CI = [0, 0.12], Akaike information criterion (AIC) = 1931.2, BIC = 2047.7. However, the fit was poorer compared to a one-factor model, χ2(54) =71.83, p = .1, RMSEA = .08 , 95% CI = [0, 0.12], AIC = 1929.9, BIC = 2034.1. According to the RMSEA cut-off values suggested by Browne and Cudeck (1993), the network model explains the data acceptable, although it performs worse than the one-factor model. Therefore, interpreting Jan’s network is not advised; we add the interpretation below for illustrative reasons, which must be read with caution. Additionally, Jan’s data demonstrated low split-half reliabilities (mid-split: r_contemp_= .37, r_temporal_= .24; odd-even split: r_contemp_= .59; r_temporal_= .45).
2. The model poorly fitted the lag-0 and lag-1 covariances, χ2(61) = 117.87, p < .001, with a root mean square error of approximation (RMSEA) = .12, 95%, CI = [0.09, 0.15], Akaike information criterion (AIC) = 2501.6, BIC = 2598.6. The fit was worse compared to a one-factor model, χ2(54) = 82.11, p = .008, RMSEA = .09 , 95% CI = [0.04, 0.12], AIC = 2479.8, BIC = 2592.3. This indicates that the network model poorly explains the data and performs worse than the one-factor model. Therefore, Ella’s network cannot be reliably interpreted. Like for Jan, the provided interpretation below should be read as a mere illustration of what could be concluded *if* the fit had been acceptable. Additionally, Ella’s data exhibited low split-half reliabilities (mid-split: *r*_contemp_= .18, *r*_temporal_ = .30; odd-even split: *r*_contemp_= .11). It was not possible to estimate the odd-even reliability of Ella's temporal network because, for the second half, all edges were estimated as zero.
3. The model fitted the lag-0 and lag-1 covariances acceptable χ2(63) = 80.54, p = .07, with a root mean square error of approximation (RMSEA) = .08, 95%, CI = [0, 0.13], Akaike information criterion (AIC) = 1674.3, BIC = 1749.2. The network model fitted better than a one-factor model, χ2(54) = 73.51 , p = .63, RMSEA = .09, 95% CI = [0.02, 0.14], AIC = 1685.3, BIC = 1776.3. This suggests that the network model adequately explains the data and performs better than the one-factor model. Interpreting the network can thus be advised. Additionally, Sam showed low split-half reliabilities (mid-split: *r*_contemp_= .20, *r*_temporal=_ .002; odd-even split (*r*_temporal_= .17). It was not possible to estimate the odd-even reliability of Sam’s contemporaneous network because, for the even half, all edges were estimated as zero.
4. The model poorly fitted the lag-0 and lag-1 covariances χ2(59) = 108.83, p <.001, with a root mean square error of approximation (RMSEA) = .22, 95%, CI = [0.15, 0.28], Akaike information criterion (AIC) = 594, BIC = 634.9. However, it fitted better than a one-factor model, χ2(54) = 111.36 , p < .001, RMSEA = .24 , 95% CI = [0.18, 0.31], AIC = 606.4, BIC = 651.8. This indicates that the network model poorly explains the data. Consequently, Kobe’s network cannot not be reliably interpreted. The following interpretation should be considered merely illustrative of what could be concluded in Kobe’s network *if* the fit were acceptable. Moreover, it was not possible to estimate the split-half reliability of Kobe’s contemporaneous and temporal networks because, for the even half, all edges were estimated as zero.
